# The role of neutrophils and G-CSF in DNFB-induced contact hypersensitivity in mice

**DOI:** 10.1002/iid3.16

**Published:** 2014-01-03

**Authors:** Anne Deen Christensen, Søren Skov, Claus Haase

**Affiliations:** 1 Department of Immunopharmacology, Novo Nordisk A/SNovo Nordisk Park, Måløv, Denmark; 2 Department of Veterinary Disease Biology, Section for Experimental Animal Models, Faculty of Health and Medical Sciences, University of CopenhagenStigbøjlen 7, Frederiksberg C, Denmark

**Keywords:** Contact hypersensitivity, neutrophil mobilization G-CSF, and neutrophils

## Abstract

Neutrophils are thought to play an important role during contact hypersensitivity (CHS) in mice, a notion which is supported by studies in which neutrophils are depleted by monoclonal antibodies (mAb). Here, we show that administration of the commonly used anti-mouse Ly6G/C mAb (clone RB6.8C5) leads to depletion of not only neutrophils but also a population of monocytes and macrophages. In contrast, depletion using a Ly6G-specific mAb (clone 1A8) only leads to depletion of neutrophils. We demonstrate that the anti-Ly6G/C mAb suppresses the inflammatory response to a higher extent than the anti-Ly6G mAb suggesting that the impact of neutrophil-depletion in the CHS model may have been overstated when based on protocols using the anti-Ly6G/C mAb. Still, the role of neutrophils in CHS is substantiated as we demonstrate that G-CSF is an important regulator of neutrophil mobilization and effector function in CHS. Indeed, G-CSF was detectable both in the inflamed tissue and in serum during the immune response and we show that blocking G-CSF results in a reduced number of neutrophils in the blood and an attenuation of the ear-swelling response in the tissue. In conclusion, this study supports that neutrophils are important drivers of inflammation in the DNFB-induced CHS model and shows that G-CSF is a significant factor in mobilizing neutrophils during the response.

## Introduction

Neutrophils are a part of the innate response to pathogens and as such regarded as the first line of defense but studies have suggested that they also possess important regulatory and like mechanisms in many inflammatory conditions. Furthermore, it has been shown that neutrophils play an important role in many autoimmune diseases and that they interact with the adaptive immune response in several ways; thus neutrophils are a potential target for new therapeutic strategies to treat autoimmune diseases in humans [1,2].

Neutrophils have been extensively studied in inflammation models in mice for example, in the collagen-induced arthritis (CIA) model [3] and K/BxN model [4] as well as in the experimental autoimmune encephalomyelitis (EAE) model [5]. In contact hypersensitivity (CHS), however, neutrophils have only been studied to a limited extent. The CHS model is an animal model where a previously sensitized animal is re-exposed to a hapten, thereby eliciting an immune reaction at the site of exposure and the model has been used as a model for human contact dermatitis [6]. In mice, contact hypersensitivity has been studied using haptens such as dinitrofluorobenzene (DNFB), FITC, and oxazolone and the response is thought to be driven mainly by T cells [7], but is known to also involve other cell types, including Langerhans cells (LC) [8], dermal dendritic cells [9], B-1 cells [10], NKT cells [11], NK cells [12], neutrophils [13], and mast cells [14]. Furthermore, several cytokines and chemokines have been implicated in the process [6]. Thus, while the CHS model in mice represents aspects of human contact dermatitis in particular, it is also relevant to study the in vivo effect of modulating different cell types and their effector mechanisms in general [6].

Previous studies have shown that depletion of neutrophils in the CHS model using the anti-mouse Ly6G/C monoclonal antibody (mAb) (clone RB6.8C5) partially reduces the ear-swelling response [13,15]. However, this antibody has recently been shown not only to deplete neutrophils but also other Ly6G/C^+^ (Gr-1^+^) cells, including subsets of monocytes and macrophages [16]. It is therefore possible that the attenuating effect obtained after administration of this antibody cannot entirely be attributed to the effect of depleting neutrophils, but may also be due to the ablation of other cell types such as monocytes and macrophages. In addition to depletion studies it has further been shown that neutrophils play a role in the early phase of the inflammation in mediating recruitment of effector CD8 T cells into the inflamed tissue [13,17,18].

In the current study, we explored the role of neutrophils in the CHS response with particular focus on the differences when using the anti-mouse Ly6G mAb (clone 1A8) compared to the anti-mouse Ly6G/C mAb (clone RB6.8C5). The results confirm that anti-Ly6G mAb specifically depletes Ly6G^+^ cells (primarily neutrophils) whereas anti-Ly6G/C mAb depletes neutrophils *and* a population of CD11b-expressing cells, which most likely constitutes monocytes and macrophages. We demonstrate for the first time that administration of anti-Ly6G/C mAb inhibits the inflammatory response in the CHS response to a higher degree than the anti-Ly6G mAb. Still, using the Ly6G-specific antibody we establish that depletion of Ly6G^+^ cells alone results in an attenuated CHS response. We further substantiate the important role of neutrophils in CHS by showing that local release of G-CSF in the tissue plays an important role in mobilization of neutrophils in the blood and that blockade of G-CSF results in a reduced number of neutrophils in the peripheral blood as well as a significantly suppressed ear-swelling response. Together, these results shed new light on the role of neutrophils in the CHS model and demonstrate that G-CSF is important for their mobilization and penetrance into the inflamed tissue.

## Results

### Increased number of neutrophils in peripheral blood and in the inflamed ear after challenge of sensitized mice

**Figure 1:**
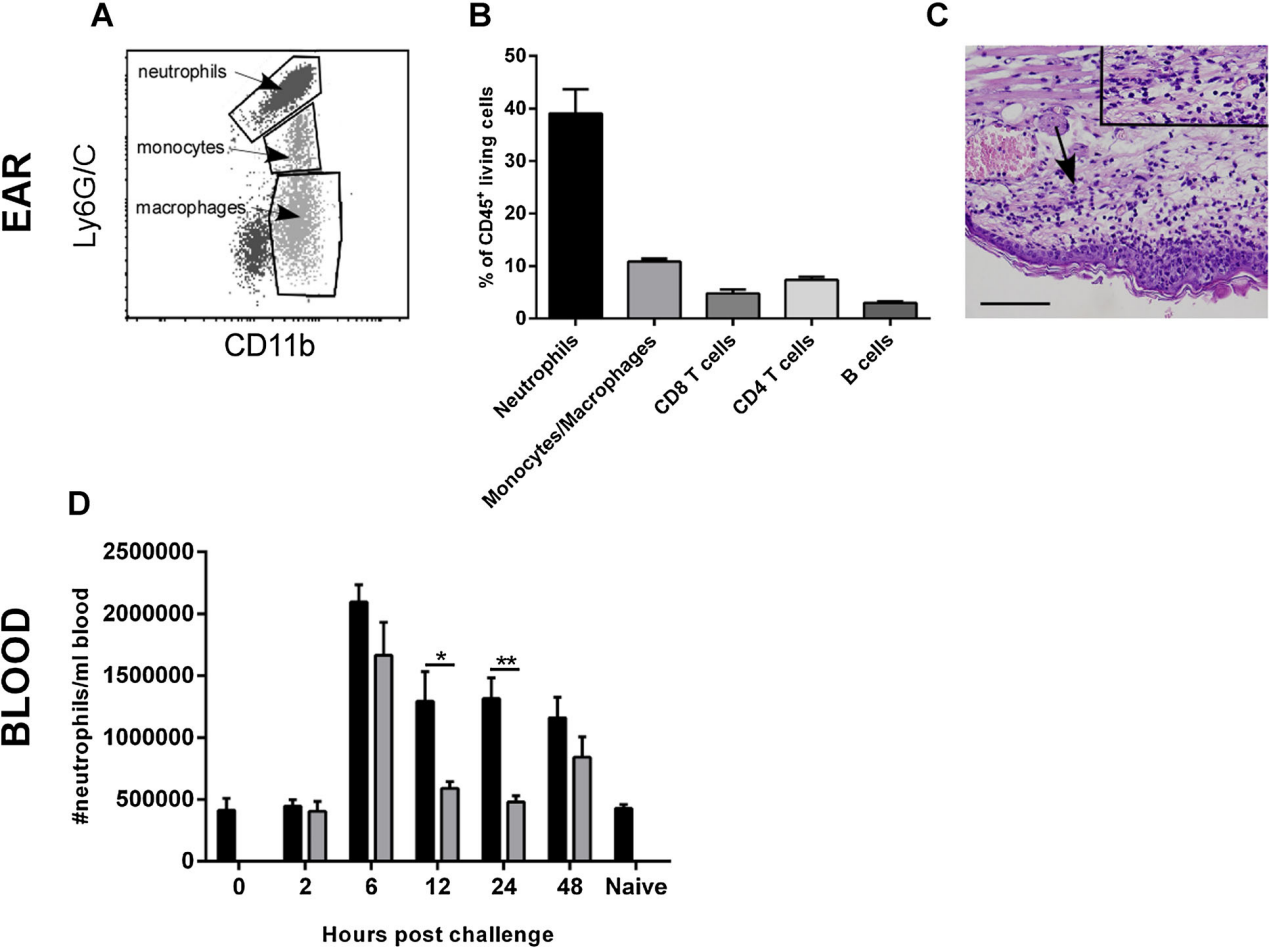
Increased number of neutrophils found both in the inflamed ear and in peripheral circulation after challenge of sensitized mice. Mice were sensitized with 0.5% DNFB and 5 days later challenged with 0.2% DNFB. Twenty-four hours after challenge ears were prepared for either histological or flow cytometric analysis of the cellular infiltrate. A: Shows a representative FACS plot of the neutrophils found in the inflamed ear 24 h after challenge. Neutrophils are defined as CD45^+^TCRβ^−^CD19^−^CD11b^+^Ly6G/C^high^ cells, monocytes as CD45^+^TCRβ^−^CD19^−^CD11b^+^Ly6G/C^intermediate^ cells and macrophages as CD45^+^TCRβ^−^CD19^−^CD11b^+^Ly6G/C^low^ cells. B: Illustrates the percentage of neutrophils, monocyte/macrophages, CD8 T cells, CD4 T cell, and B cells, respectively out of the entire population of CD45^+^ living cells in the inflamed ear 24 h after challenge. C: Hematoxylin & eosin (HE) staining of a DNFB-challenged ear 24 h after challenge shows heavy infiltration of polymorphonuclear cells (PMNs) (arrow). An enlarged image of the PMNs pointed out by the arrow is inserted in the upper right corner. Scale bar corresponds to 0.1 mm. D: Blood from sensitized- (black bars) and non-sensitized (grey bars) groups were collected at different time points after challenge and subsequently the absolute number of neutrophils/mL blood was quantified using flow cytometry and BDTrucount beads. Data are depicted as mean ± SEM, *P < 0.05, **P < 0.01 and ***P < 0.001. *n* = 5/group. A–C depict one out of several representative experiments. Blood samples in (D) were confirmed by cell count acquired using a Medonic Hematology Analyzer.

To confirm the presence of neutrophils during a CHS response in mice, the cellular content of inflamed ears was analyzed by flow cytometry and histology 24 h after DNFB-challenge in sensitized mice (Fig. 1A–C). Neutrophils were gated as CD45^+^TCRβ^−^CD19^−^CD11b^+^Ly6G/C^high^ and are shown as the Ly6G/C^high^-population in Figure 1A. The plot furthermore depicts the CD45^+^TCRβ^−^CD19^−^CD11b^+^Ly6G/C^intermediate^ monocyte-population and the CD45^+^TCRβ^−^CD19^−^CD11b^+^Ly6G/C^low^-population of macrophages gated as described in [16,19]. When quantified, neutrophils constituted by far the most abundant cell type with around 40% of the infiltrating cells in the inflamed tissue (Fig. 1B). These findings were confirmed by histological analysis on sections of inflamed ear tissue excised 24 h after challenge which demonstrates heavy infiltration of polymorphonuclear cells (PMNs), some of which are accumulated in intraepithelial micro abscesses (Fig. 1C).

Next, the absolute number of neutrophils in the blood was measured at different time-points after challenge of previously sensitized or non-sensitized mice. As seen in Figure 1D neutrophilia was present 6 h after challenge in sensitized and challenged mice (black bars) as well as in mice only challenged without previous sensitization (grey bars). The observation that neutrophilia was present in both groups at the earliest time-points, is most likely a response independent of previous priming, however, at 12 and 24 h the number of neutrophils was only sustained at increased levels in mice that were both sensitized and challenged. Based on these findings, we conclude that neutrophils are involved in the inflammatory process in contact hypersensitivity both locally in the inflamed tissue as well as systemically in peripheral blood.

### Depletion of neutrophils using anti-Ly6G/C or Ly6G-specific antibodies

**Figure 2:**
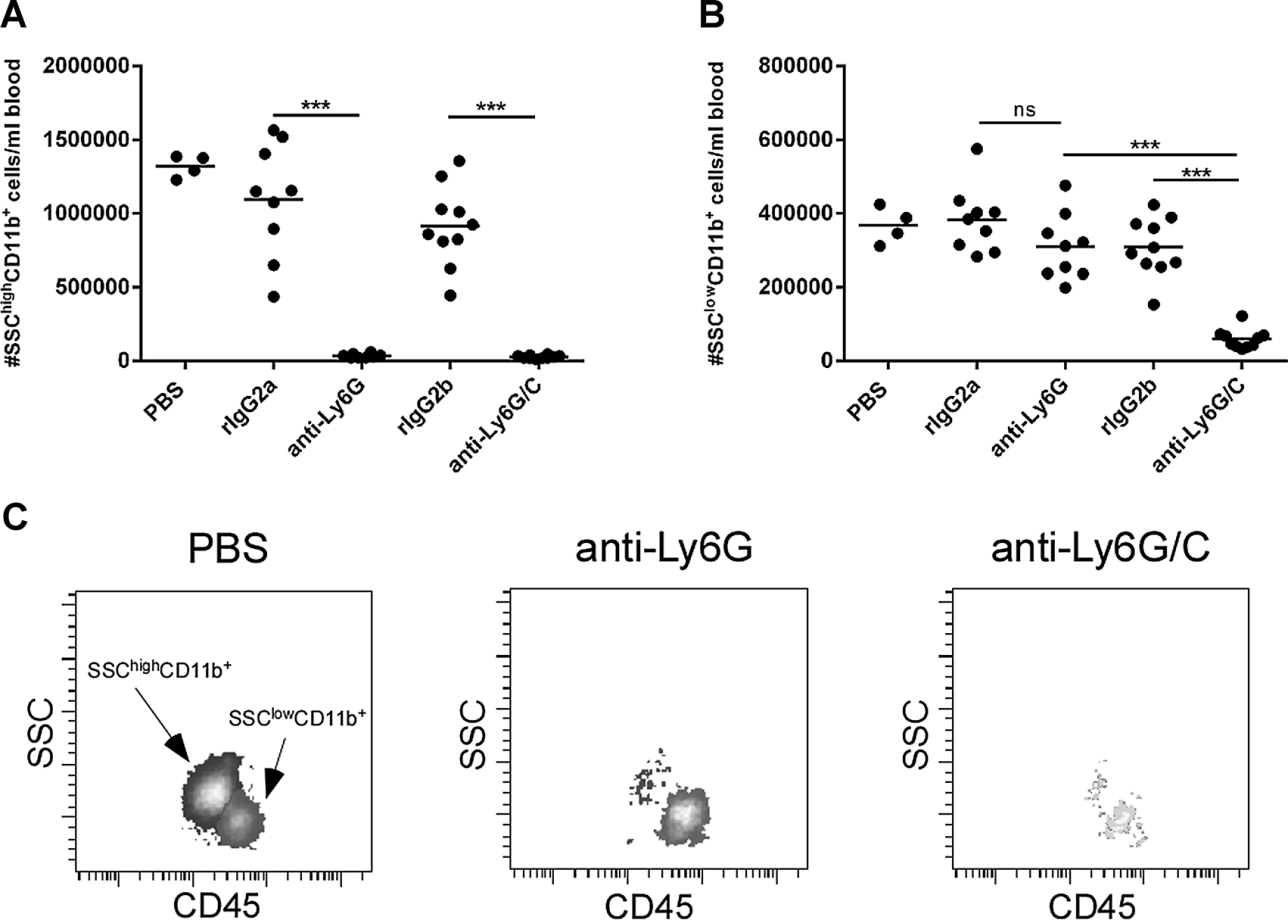
Differences in depletion efficacy after administration of two different neutrophil-depleting antibodies. Blood samples were taken 24 h after administration with either PBS, rIgG2a (isotype control for anti Ly6G mAb), rIgG2b (isotype control for anti-Ly6G/C mAb), anti-Ly6G mAb (clone 1A8), or anti-Ly6G/C mAb (clone RB6.8C5), stained for relevant markers and analyzed by flow cytometry. The depleting antibodies and isotype controls were injected in 1 mg/mouse. Absolute number (#) of neutrophils and monocytes was quantified using BDTrucount beads. A: Absolute number of neutrophils defined as #CD45^+^TCRβ^−^CD19^−^CD11b^+^SideScatter(SSC)^high^ cells/mL blood (“SSC^high^CD11b^+^”). B: Absolute number of monocytes defined as #CD45^+^TCRβ^−^CD19^−^CD11b^+^SSC^low^ cells/mL blood (“SSC^low^CD11b^+^”). C: Representative FACS plots for the three groups of animals treated with PBS, anti-Ly6G mAb or anti-Ly6G/C mAb, respectively. The plots show CD11b^+^ cells. Data are depicted as mean ± SEM, *n* = 4–10/group, *P < 0.05, **P < 0.01 and ***P < 0.001. The figure depicts one out of two representative experiments.

Previous studies investigating the effect of neutrophil-depletion in the CHS model have used the anti-Ly6G/C mAb (clone RB6.8C5) [13,15]. In addition to Ly6G, this antibody also binds to Ly6C (which in addition to neutrophils, is mainly present on monocytes [20]) and the antibody can therefore not be considered completely neutrophil-specific. In contrast, a Ly6G-specific mAb (clone 1A8) has been described which more specifically depletes neutrophils in vivo [16]. To confirm that different cell subsets are being depleted by the two antibodies, both antibodies and their respective isotype controls were administered in groups of sensitized mice one day prior to challenge in 1 mg/mouse. Depletion was analyzed by flow cytometry on the day of challenge (the day after administration of the antibody) to determine the degree of depletion by the two antibodies. To avoid competition with the depleting antibody, Ly6G was not used as an analytical marker to identify neutrophils; instead neutrophils were gated as CD45^+^TCRβ^−^CD19^−^CD11b^+^SSC^high^ and the absolute number of the relevant subsets of cells was quantified with BDTrucount beads. Figure 2A,B depict the absolute numbers of CD11b^+^SSC^high^ cells (A) and CD11b^+^SSC^low^ cells (B) and show that the anti-Ly6G/C mAb depleted neutrophils (CD11b^+^SSC^high^) as well as a fraction of CD11b^+^SSC^low^ cells which most likely constitutes monocytes. In contrast, anti-Ly6G mAb exclusively depleted the CD11b^+^SSC^high^ population and not the CD11b^+^SSC^low^ population. The representative FACS-plots in Figure 2C depict the CD11b^+^ population and illustrate the reduction in the population of neutrophils (SSC^high^) in mice treated with either anti-Ly6G mAb or anti-Ly6G/C mAb, respectively compared to a PBS-treated mouse. Additionally, a decline in the monocyte-population (SSC^low^) appears in the mouse treated with anti-Ly6G/C mAb which was not seen in neither of the other two groups. Taken together, these data confirm the enhanced specificity for neutrophils of the anti-Ly6G mAb compared to the anti-Ly6G/C mAb.

### Depletion of neutrophils using anti-Ly6G monoclonal antibody attenuates the ear-swelling response in the DNFB-induced CHS-model

**Figure 3:**
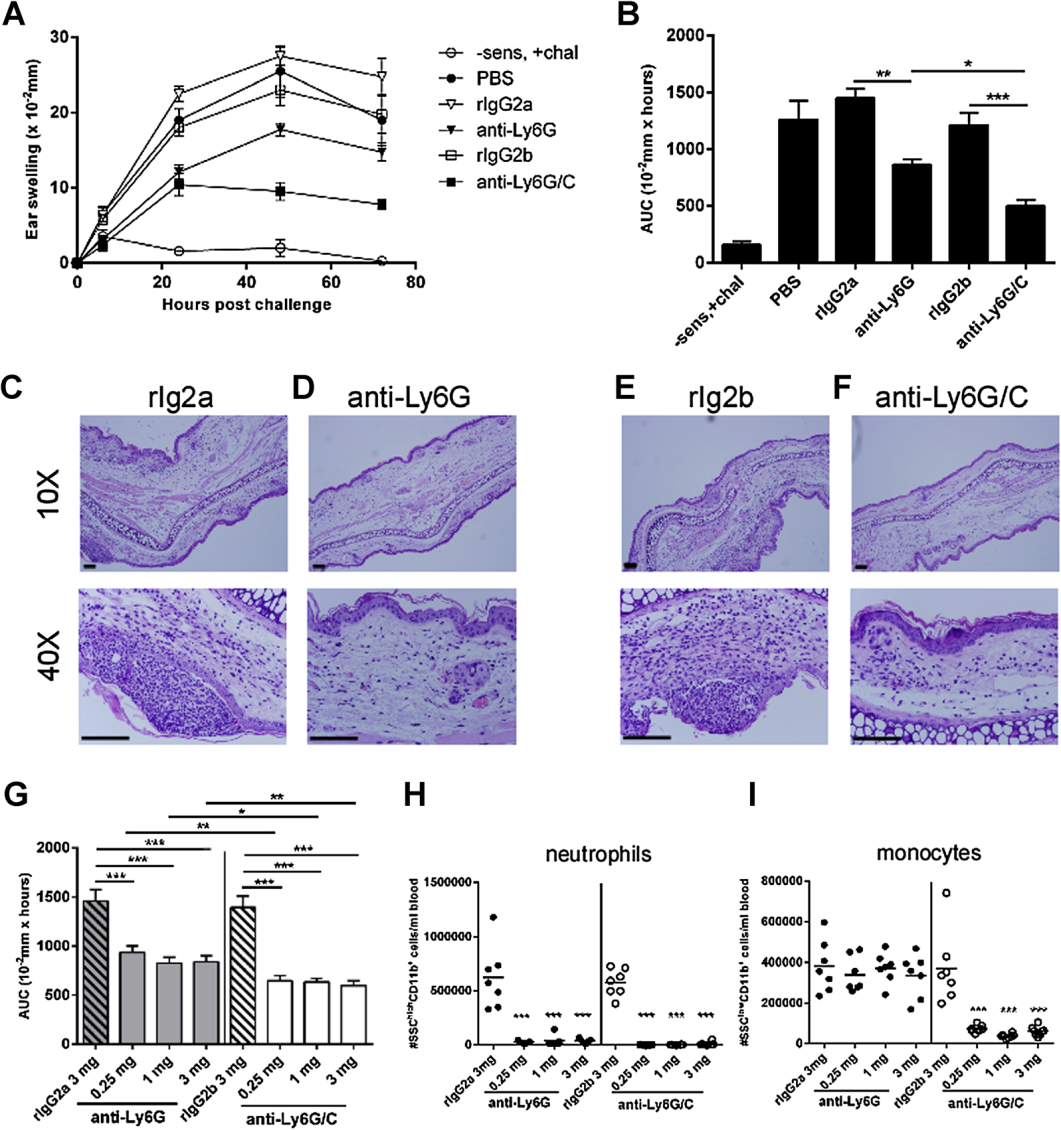
Depletion of Ly6G/C^+^ cells inhibits the ear-swelling response to a higher extent than depletion of Ly6G^+^ cells alone. Mice were sensitized with 0.5% DNFB (except the −sens, +chal group) and 5 days later all groups were challenged with 0.2% DNFB. The day before challenge, groups were treated with 200 μL PBS, 1 mg/mouse rIgG2a (isotype control for anti-Ly6G mAb), 1 mg/mouse rIgG2b (isotype control for anti-Ly6G/C mAb), 1 mg/mouse anti-Ly6G mAb (clone 1A8), or 1 mg/mouse anti-Ly6G/C mAb (clone RB6.8C5), respectively and ear swelling was measured 0–72 h post-challenge. A: Ear-swelling response 0–72 h after challenge in the respective groups. B: Area under curve (AUC). C–F: Representative histological sections after hematoxylin and eosin (HE)-staining from a rIgG2a (C), anti-Ly6G mAb (D), rIgG2b (E) and an anti-Ly6G/C mAb-treated mouse (F) 24 h post-challenge. The upper row includes images from a 10× magnification and the lower row includes images from a 40× magnification. Scalebar = 0.1 mm. G: Anti-Ly6G mAb and anti-Ly6G/C mAb were tested in 0.25, 1 and 3 mg/mouse, respectively together with their isotype controls tested in 3 mg/mouse. All compounds were injected 1 day prior to challenge and ear swelling was measured 0–72 h after challenge. Shown is ear swelling as area under curve (AUC). H and I: Degree of depletion was performed on the day of challenge (Day 0) by flow cytometry on all groups and the absolute number (#) of SSC^high^CD11b^+^ cells (H) and SSC^low^CD11b^+^ cells (I) was estimated. Data are depicted as mean ± SEM, *n* = 5/group (except in G–I where *n* = 8/group), *P < 0.05, **P < 0.01, and ***P < 0.001.

To investigate if administration of the anti-mouse Ly6G/C mAb had a different impact on the CHS response than the anti-mouse Ly6G mAb, the two antibodies and their respective isotype controls were administered in groups of sensitized mice one day prior to challenge at a dose of 1mg/mouse. Figure 3A,B shows the ear-swelling response in the different groups 0–72 h after challenge (A) and as area under curve (AUC) (B). The figure demonstrates that administration of both anti-Ly6G/C mAb and anti-Ly6G mAb resulted in a significantly suppressed ear-swelling response compared with their respective isotype controls. Furthermore, the anti-Ly6G/C mAb suppressed the ear-swelling response to a significantly higher extent than the anti-Ly6G mAb suggesting that depletion of both neutrophils and monocytes (Fig. 3A,B) has a bigger impact on the ear-swelling response than depletion of neutrophils alone. The effect seen on ear swelling was confirmed by histology (see Fig. 3C) and confirmed that in the anti-Ly6G mAb- and anti-Ly6G/C mAb-treated mice, respectively infiltration of inflammatory cells and especially infiltration of PMNs was less pronounced and ear thickness was clearly reduced compared to controls. Furthermore, in the isotype control-treated mice, PMNs accumulate in intraepithelial foci as shown in the lower row in C and E - something that was not observed in the mice treated with anti-Ly6G- or anti-Ly6G/C mAb.

To assess whether the different effect of the two antibodies could be explained by a dosing effect, both antibodies were tested in three different doses administered 1 day prior to challenge. However, as shown in Fig. 3G, no significant dosing effect of anti-Ly6G mAb was observed and at all three doses of antibodies, anti-Ly6G/C mAb reduced the ear-swelling response more efficiently than anti-Ly6G mAb. Further, flow cytometric analysis confirmed that neutrophils were completely depleted by all doses of anti-Ly6G mAb or anti-Ly6G/C mAb (Fig. 3H) and additionally demonstrated that all three doses of anti-Ly6G/C mAb depleted a fraction of SSC^low^CD11b^+^ cells (monocytes, Fig. 3I) as seen previously (see Fig. 2). Overall, we conclude that depletion with anti-Ly6G/C mAb or anti-Ly6G mAb 1 day prior to challenge resulted in a reduced ear-swelling response and that the anti-Ly6G/C mAb suppressed the response to a higher extent than the anti-Ly6G mAb.

### Differential impact on inflammatory cytokines and cellular infiltration after administration of anti-Ly6G/C mAb or anti-Ly6G mAb

**Figure 4:**
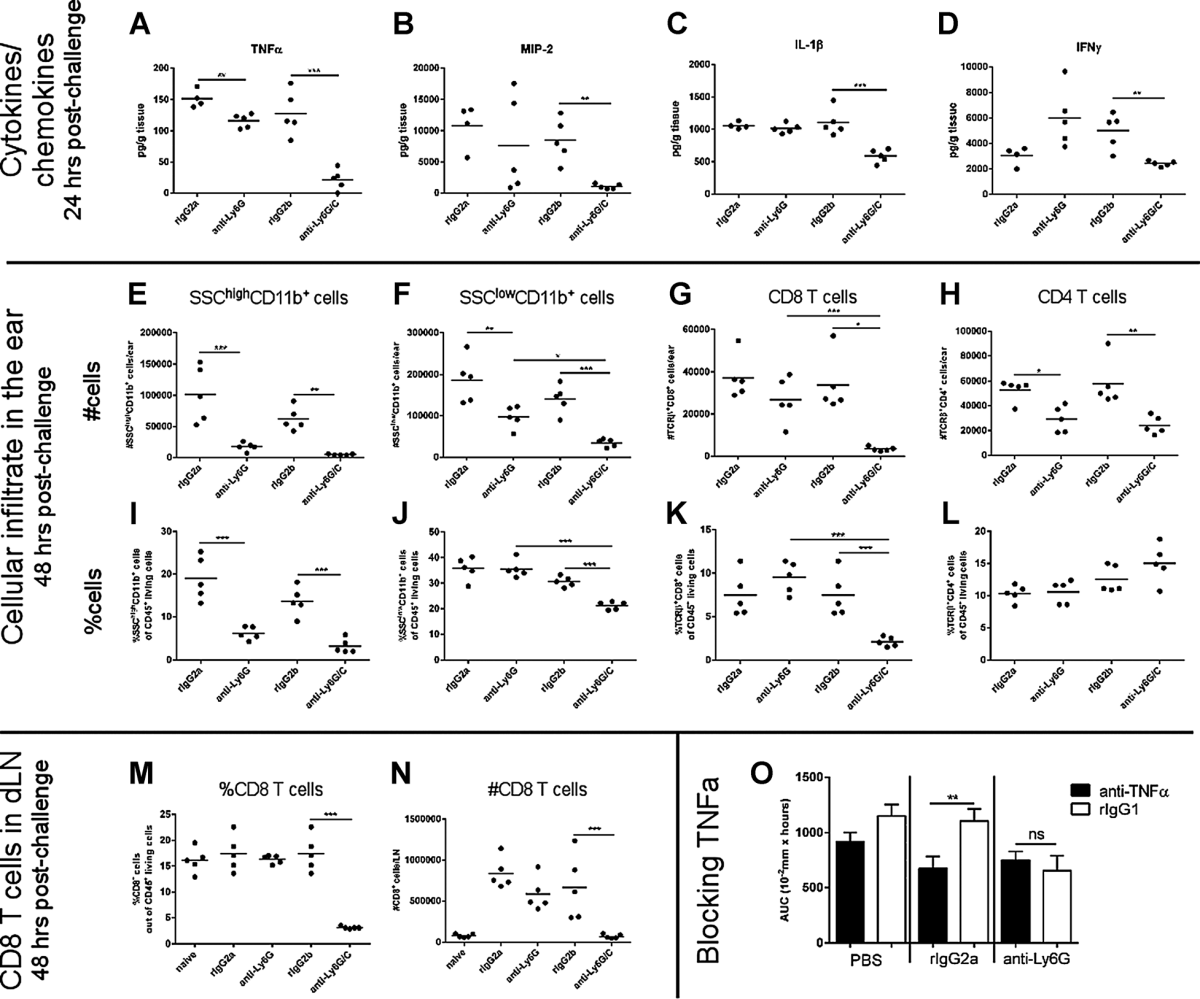
Differences in the inflammatory reaction locally in the ear and in the draining lymph node between treatments with anti-Ly6G/C mAb versus anti-Ly6G mAb. A–F: Homogenates of ear tissue were prepared 24 h after challenge and analyzed for their content of a number of selected cytokines and chemokines. Shown are the concentrations of TNFα (A), MIP-2 (B), IL-1β (C), and IFNγ (D). E–L: Cells infiltrating the inflamed ear were isolated, stained for relevant markers and analyzed by flow cytometry 48 h after challenge. Granulocytes were gated as CD45^+^SSC^high^CD11b^+^ cells and monocyte-like cells were gated as CD45^+^SSC^low^CD11b^+^ cells. E: Absolute number (#) of granulocytes/ear, I: %granulocytes out of CD45^+^ living cells, F: Absolute number (#) of monocyte-like cells/ear, J: %monocyte-like cells out of CD45^+^ living cells, G: Absolute number (#) of CD8 T cells/ear, K: %CD8 T cells out of CD45^+^ living cells. H: Absolute number (#) of CD4 T cells/ear, L: %CD4 T cells out of CD45^+^ living cells. Cells from the ear draining lymph nodes (dLN) were isolated, stained for relevant markers and analyzed for their content of CD8 T cells by flow cytometry 48 h after challenge. M: Absolute number (#) of CD8 T cells in the ear dLN. N: %CD8 T cells out of CD45^+^ living cells in the ear dLN. O: A rat anti-mouse TNFα mAb or its isotype control rIgG1 were administered in a dose of 0.5 mg/mouse in groups of mice treated at the same time with 1 mg/mouse anti-Ly6G mAb, 1 mg/mouse rIgG2a or 200 μL PBS, respectively a day prior to challenge. Ear swelling was measured 0–72 h after challenge and area under curve (AUC) is shown. Data are depicted as mean ± SEM, *n* = 5/group, *P < 0.05, **P < 0.01 and ***P < 0.001. A–L depicts one out of two representative experiments.

Having demonstrated a difference in the ear-swelling response after depletion of neutrophils we investigated the inflammatory reaction locally in the tissue in further details. Firstly, homogenates of inflamed ear-tissue from anti-Ly6G mAb-, anti-Ly6G/C mAb- or isotype-control treated animals were analyzed for their content of selected neutrophil-related cytokines and chemokines 24 h after challenge. As shown in Figure 4A–D the levels of TNFα, MIP-2, IL-1β and IFNγ were significantly reduced in the anti-Ly6G/C mAb-treated group compared to isotype-control treated mice 24 h after challenge. The levels of MCP-1, KC, MIG, LIX, IL-4, IL-6, IL-10, RANTES, and G-CSF were not significantly changed in any of the treatment groups (data not shown). In contrast, administration of anti-Ly6G mAb only resulted in a slightly reduced level of TNFα and not any of the other selected analytes. Secondly, infiltrating cells were isolated from the inflamed ear 48 h after challenge, stained for relevant markers and analyzed by flow cytometry. In Figure 4E, it is shown that administration of both anti-Ly6G mAb and anti-Ly6G/C mAb led to depletion of granulocytes (SSC^high^CD11b^+^ cells) in the inflamed ear tissue. The population of SSC^low^CD11b^+^ cells was significantly suppressed in both depletion groups compared to their isotype controls when given as absolute number (#). However, anti-Ly6G/C mAb reduced both the percentage and absolute number of SSC^low^CD11b^+^ cells in the inflamed ear to a significantly higher extent compared to anti-Ly6G mAb. Furthermore, administration of anti-Ly6G/C mAb resulted in a decline in infiltration of CD8 T cells both in percentage as well as in absolute number (Fig. 4G,K)—something which was not seen after treatment with anti-Ly6G mAb and suggests that anti-Ly6G/C mAb affects infiltration of monocytes as well as CD8 T cells whereas depletion using anti-Ly6G mAb only affects monocytes and to a lesser extent than the anti-Ly6G/C antibody. Both antibodies had a moderate effect on the number of CD4 T cells in the inflamed tissue. Having observed a difference in the infiltration of CD8 T cells between the two antibodies we further wanted to investigate potential differences in the number of CD8 T cells in the ear draining lymph node (dLN). Administration of anti-Ly6G/C mAb resulted in a significantly lower number and percentage of CD8 T cells in the dLN 48 h after challenge while anti-Ly6G mAb did not affect the presence of CD8 T cells (Fig. 4M,N). This again emphasizes the different impact the two antibodies exert on the inflammatory mediators during the CHS response.

As depicted in Figure 4A anti-Ly6G/C reduced TNFα-levels in the inflamed tissue, whereas anti-Ly6G mAb only did that to a limited extent. Thus, we wanted to examine whether blocking TNFα could reduce the ear-swelling response in the anti-Ly6G mAb-injected mice even further. However, as can be seen from Figure 4O blocking TNFα did not reduce the ear swelling any further in the anti-Ly6G mAb-treated mice whereas TNFα-blockade clearly had an effect in non-depleted mice as shown by others [21]. This suggests that TNFα is dispensable in anti-Ly6G mAb-treated mice and cannot solely explain the different efficacy of anti-Ly6G mAb versus anti-Ly6G/C mAb.

Taken together, we conclude that anti-Ly6G/C mAb suppresses the inflammation to a higher extent than the anti-Ly6G mAb both in regard to release of cytokines and chemokines, infiltration of CD8 T cells and monocyte-like cells in the inflamed ear as well as presence of CD8 T cells in the ear dLN.

### No further effect of anti-Ly6G mAb-mediated depletion during the sensitization phase

**Figure 5:**
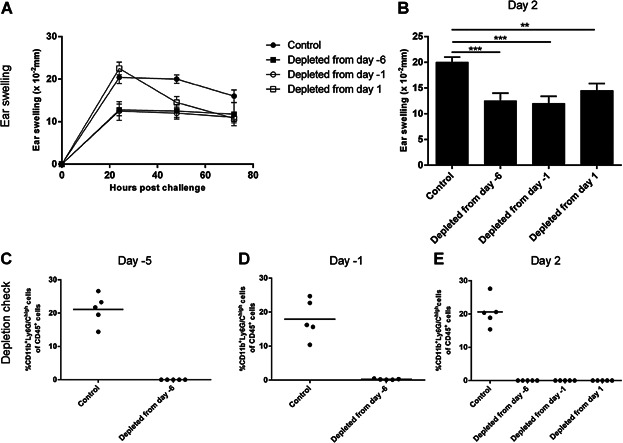
No further effect of anti-Ly6G mAb-mediated depletion during the sensitization phase. Previously sensitized groups of mice were challenged with DNFB and treated with anti-mouse Ly6G mAb (clone 1A8) in 1 mg/mouse intraperitoneally starting either the day before sensitization (treated Day −6, −1 and 1), the day before challenge (treated Day −1 and 1) or the day after challenge (treated Day 1 only). One group received 200 μL PBS on Day −6, −1, and 1 as a control. A: Ear-swelling response 0–72 h post-challenge in the control group and in the groups depleted from Day −6, −1 or 1, respectively. B: Ear swelling 2 days after challenge. C–E: Depletion was confirmed by flow cytometry on Day −5 (the day of sensitization) (C), Day −1 (a day prior to challenge) (D) and again on Day 2 (E) to verify that neutrophils in the different groups were sufficiently depleted. Neutrophils were gated as CD45^+^CD11b^+^Ly6G/C^high^ cells and quantified in % out of CD45^+^ cells. Data are depicted as mean ± SEM, *n* = 5/group, *P < 0.05, **P < 0.01, and ***P < 0.001. The figure depicts one out of two representative experiments.

Next, we wanted to determine whether depletion of neutrophils both during the sensitization and challenge phase would lead to a stronger suppression of the CHS response than neutrophil-depletion during the challenge-phase alone. To address this question, neutrophils were depleted on Day −6, −1 and 1 (i.e., from one day prior to sensitization and onwards), on Day −1 and 1 (i.e., from one day prior to challenge and onwards) or solely on Day 1 (i.e., the day after challenge). Depletion was confirmed throughout the experiment (Fig. 5C–E). As shown in Figure 5 depletion of neutrophils one day prior to sensitization (Day −6) reduced ear-swelling response to the same extent as depletion 1 day prior to challenge (Day −1) suggesting that the main effect of neutrophil-depletion is due to a role during the challenge phase. Furthermore, depletion 24 h after challenge (Day 1) reduced the ear-swelling response significantly after 48 h (B) confirming that neutrophils also play a role in sustaining the inflammation. Based on this study, we conclude that depletion of neutrophils only during the challenge phase suppresses the ear-swelling response to the same extent as when neutrophils are absent both during the sensitization- and challenge-phase. This suggests that neutrophils contribute to the inflammatory response mainly during the challenge phase.

### G-CSF is produced locally during CHS and is released into circulation

**Figure 6:**
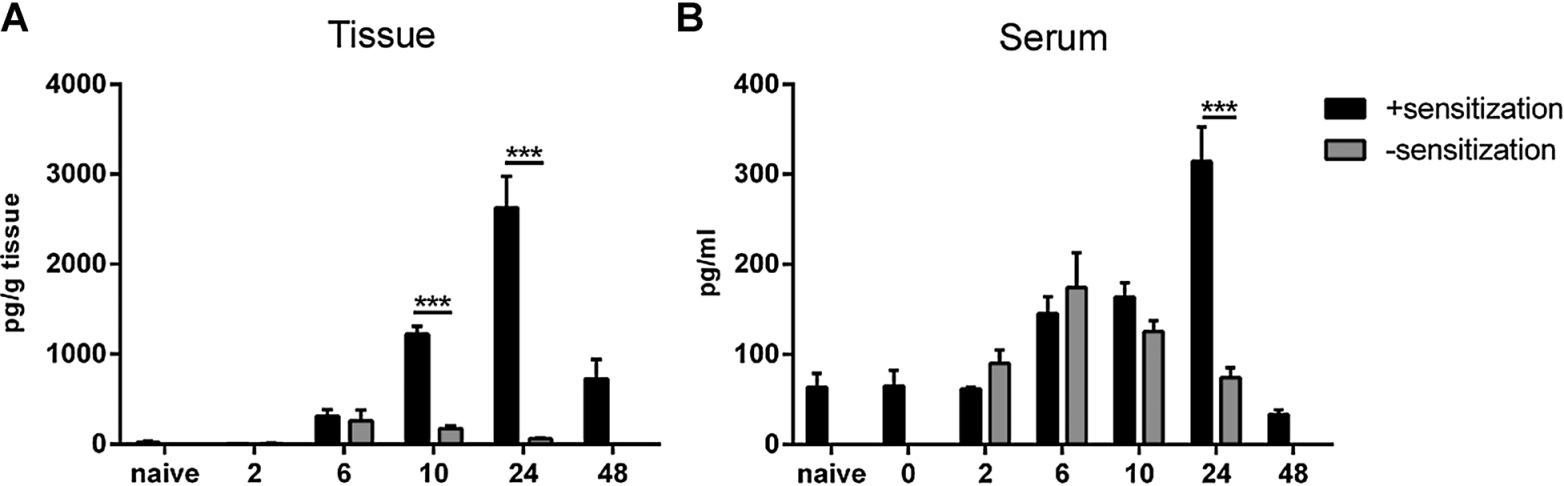
G-CSF is produced locally in the inflamed ear-tissue after challenge and is released into the circulation. Serum samples and homogenates of inflamed ear tissue were prepared 2-, 6-, 10- and 24 h after challenge in non-sensitized groups (grey bars), 0, 2, 6, 10, 24, and 48 h after challenge in sensitized groups as well as in a non-treated naive group. Levels of G-CSF were analyzed by ELISA. A: Levels of G-CSF in inflamed ear tissue in pg/g tissue. B: Levels of G-CSF in serum in pg/mL. Data are depicted as mean ± SEM, *n* = 5/group, *P < 0.05, **P < 0.01, and ***P < 0.001. The figure depicts one out of two representative experiments.

Having established an important role of neutrophils, we wanted to investigate if G-CSF was involved in CHS in mice as G-CSF is an important mediator of neutrophil mobilization from the bone marrow into circulation [22]. Firstly, we measured levels of G-CSF by ELISA in homogenates of inflamed ear tissue as well as in serum at different time points during the CHS response. Figure 6A shows that in sensitized and challenged mice (black bars) the level of G-CSF in the inflamed ear tissue was significantly up-regulated. Further, G-CSF could not be detected in non-inflamed naive ear tissue and was expressed at lower levels in challenged ear-tissue from non-sensitized mice (grey bars). Moreover, G-CSF peaked in serum approximately 24 h after challenge and was higher and more pronounced in animals which were both sensitized and challenged (black bars) compared to non-sensitized animals (grey bars) both after 10 and 24 h (Fig. 6B). Taken together, we conclude that G-CSF is present in the tissue after exposure to the hapten and can be detected at elevated levels in serum.

### G-CSF neutralization ameliorates the ear-swelling response and lowers the number of neutrophils in peripheral blood

**Figure 7:**
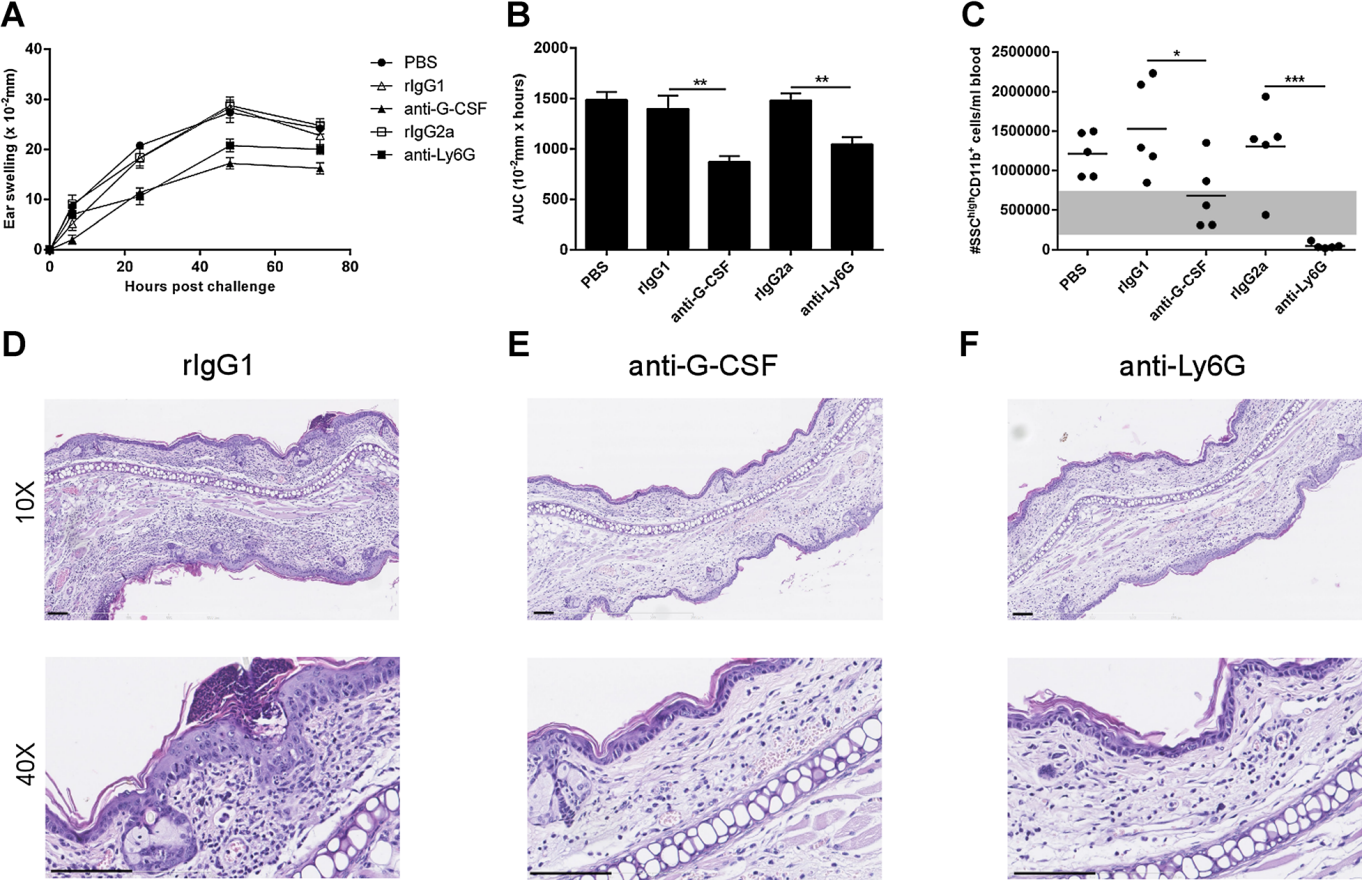
Blocking G-CSF leads to a suppressed ear-swelling response and decreased number of neutrophils in peripheral blood. Mice were sensitized with 0.5% DNFB and challenged 5 days later with 0.2% DNFB. Two hours prior to challenge, mice were treated with PBS, 0.5 mg rIgG1 (isotype control for the anti-G-CSF mAb), 0.5 mg anti-G-CSF mAb, 1 mg rIgG2a (isotype control for anti-Ly6G mAb) or 1 mg anti-Ly6G mAb (clone 1A8), respectively. Ear swelling was measured 6, 24, 48, and 72 h after challenge as shown in A. B: Ear-swelling response summarized as area under curve (AUC). C: Blood samples were taken 24 h after challenge, stained for relevant markers and analyzed by flow cytometry. Number of neutrophils was quantified using BDTrucount beads and defined as TCRβ^−^CD19^−^CD11b^+^SSC^high^ cells. Absolute number (#) of neutrophils/mL blood in the different groups. The shaded area illustrates neutrophil level in blood of naive mice. D–F: Representative histological sections after hematoxylin and eosin (HE)-staining from a rIgG1 (D), an anti-G-CSF mAb (E) and an anti-Ly6G mAb-treated mouse (F) 24 h post-challenge. The upper row shows images with a 10× magnification and the lower row shows images with 40× magnification. Scalebar = 0.1 mm. Data are depicted as mean ± SEM, *n* = 5/group, *P < 0.05, **P < 0.01 and ***P < 0.001. The figure depicts one out of two representative experiments.

To determine the importance of G-CSF during contact hypersensitivity we administered a blocking G-CSF antibody to animals 2 h prior to challenge in a dose of 0.5 mg/mouse and compared it with its isotype control rIgG1 in a similar dose. As demonstrated in Figure 7A, blocking G-CSF led to a significantly reduced ear-swelling response compared to isotype-control treatment and the inhibition was comparable to depletion of neutrophils using the anti-Ly6G mAb. Furthermore, blockade of G-CSF led to a significant reduction in the absolute number of neutrophils in blood compared to its isotype control measured 24 h after challenge (Fig. 7C). To confirm the observations seen on the ear-swelling response, sections of ear tissue were prepared for histology from the different groups 24 h after challenge. In Figure 7D–F representative histology sections stained with HE are shown from isotype control (D), anti-G-CSF mAb (E) and anti-Ly6G mAb (F) treated mice, respectively. From these it can be seen that both blocking G-CSF (E) and depletion of neutrophils (F) resulted in a reduced infiltration of inflammatory cells especially in dermis compared to an isotype-control treated mouse (D). Taken together, these data demonstrate that G-CSF is important for a full-blown CHS response and that neutralization of G-CSF reduces the number of neutrophils in circulation during CHS.

## Discussion

In this study, we investigate the role of neutrophils in the DNFB-induced CHS model and we show for the first time that specific depletion of neutrophils (using an anti-Ly6G mAb) leads to a reduced CHS response. Our results also show that, in addition to neutrophil infiltration in the inflamed tissue, increased numbers of neutrophils can be detected in the peripheral blood during the CHS response; a finding that has previously been described in other neutrophil-mediated animal inflammation models, but not in the CHS model [23]. Finally, we demonstrate that G-CSF is an important factor during contact hypersensitivity since blockade of G-CSF results in an ameliorated inflammatory response.

Contact hypersensitivity in mice is driven mainly by CD8^+^ T cells whereas CD4^+^ T cells are thought to mainly regulate the response via regulatory T cells [6,24,25]. Still, neutrophils are very abundant in the inflamed tissue and constitute the majority of the infiltrating cells supporting the interpretation that they are important effector cells in the tissue. Few studies have addressed the role of neutrophils in the CHS model. Firstly, a study by Dilulio et al. [15] suggested that immediately after challenge neutrophils are recruited into the tissue where they subsequently mediate further infiltration of hapten-specific CD8 T cells. This was confirmed by a second study showing that the intensity of neutrophil influx directly affected the magnitude of effector T cell infiltration [13]. Secondly, Kish et al. [17] have investigated the CD8 T cell–neutrophil interaction in the early phase of contact hypersensitivity and showed that a small number of IFNγ- and IL-17-producing CD8 T cells are recruited into the tissue rapidly after challenge and subsequently drive the succeeding CXCL1- and CXCL-2-mediated neutrophil infiltration. Additionally, the same group demonstrated that the subsequent neutrophil-mediated recruitment of hapten-specific CD8 T cells is dependent on expression of Fas-ligand and perforin on neutrophils [18]. In most studies, depletion of neutrophils has been mediated by using the anti-Ly6G/C mAb (clone RB6.8C5). However, as it was recently demonstrated that this antibody not only depletes neutrophils but also a fraction of Gr1^+^ monocytes and macrophages [16], we have compared depletion by anti-Ly6G/C mAb with an anti-Ly6G mAb (clone 1A8) with a greatly improved specificity for neutrophils [16]. We confirm that the anti-Ly6G/C mAb depletes neutrophils but we also show that it partially depletes a population of CD11b^+^SSC^low^ cells which is thought to contain monocytes whereas the anti-Ly6G mAb only depletes neutrophils. Administration of either of the antibodies results in a significantly suppressed hapten-induced inflammation but suppression is most pronounced in the anti-Ly6G/C mAb-treated animals. These data confirm that neutrophils play a role in contact hypersensitivity but also show that depletion using the anti-Ly6G/C mAb most likely overestimates the relative importance of neutrophils in this model. Furthermore, our data are the first to show the impact of neutrophils in the CHS model by neutrophil-specific depletion with the anti-Ly6G mAb. Treatment with the anti-Ly6G/C mAb resulted in reduced release of TNFα, IFNγ, MIP-2, and IL-1β locally in the tissue together with a reduced infiltration of CD8 T cells which was not observed with specific depletion of neutrophils with anti-Ly6G mAb. Together, this may indicate that the fraction of monocytes, presumably turning into tissue-macrophages when recruited into the inflamed tissue, may have a significant impact on the inflammatory response with respect to both cytokine/chemokine induction and infiltration of effector cells—a hypothesis that will need further investigation. The reason for the reduced number of CD8 T cells both in the dLN and in the challenged ear after administration of anti-Ly6G/C mAb requires further studies but may suggest that depletion of the Gr1^+^ monocyte population (SSC^low^CD11b^+^-cells) affects the effector population of CD8 T cells but whether this is a direct or indirect effect remains to be elucidated.

However, this study also highlights that depletion of neutrophils only results in a partial reduction of the response and that infiltration of hapten-specific CD8 T cells is not completely dependent on the infiltration of neutrophils as suggested [18]. Still, the presence of neutrophils probably boosts the local inflammatory milieu, which facilitates additional recruitment of leukocytes, including CD8 T cells.

This is in accordance with our data demonstrating that depletion of neutrophils 48 h after challenge significantly suppresses the established inflammation, indicating that neutrophils also play a role in sustaining the response and are not only important in the early-phase immediately after challenge. This would be in agreement with other animal models of inflammation, including the collagen-induced arthritis-model, where depletion of neutrophils on the peak of the response ameliorates established arthritis [3].

To further understand how neutrophils are mobilized during inflammation we focused on the role of G-CSF in the CHS response and demonstrate that levels of G-CSF are increased both in the inflamed tissue as well as in serum. We also showed that neutralization of G-CSF reduced the number of neutrophils in the peripheral blood during contact hypersensitivity and suppressed the ear-swelling response to a similar extent as neutrophil depletion. Nevertheless, when analyzing the cellular infiltrate in the inflamed ear, neutrophils could still be detected suggesting that neutralization of G-CSF could not completely prevent neutrophil influx into the tissue (data not shown). This is in accordance with the hypothesis that G-CSF is thought to be neither chemotactic nor chemokinetic but mainly responsible for mobilization of neutrophils from the bone marrow [26]. Thus, neutralization of G-CSF does not prevent the remaining circulating neutrophils to enter the inflamed ear tissue but rather inhibits neutrophilia in circulation. Apart from neutrophil mobilization from the bone-marrow, G-CSF also has other functions including activation of endothelial cells [27], modulation of leukocyte adhesion molecules [28,29], enhancing angiogenesis [30], induction of the neutrophil chemoattractants CXCL5 and CXCL6 [28], and prolongation of neutrophil survival [31,32]. Additionally, it is suggested that G-CSF increases the expression of CD44 [23] and CD11b [33] on neutrophils but decreases their expression of CD62L (L-selectin) [33]. Thus, apart from a direct effect on neutrophil mobilization from the bone marrow, neutralization of G-CSF may also influence other aspects of the CHS response.

Taken together, our study demonstrates that neutrophils are important during the CHS response and that G-CSF plays a role in mobilizing neutrophils into circulation. Furthermore, neutralization of G-CSF results in a reduced number of neutrophils in peripheral blood during the inflammatory response as well as a suppressed ear-swelling response. Finally, our data demonstrate that treatment with an anti-Ly6G antibody in the CHS model results in a more specific neutrophil-targeted depletion and therefore confirms a role for these cells in contact hypersensitivity.

## Materials and Methods

### Mice

Female BALB/c mice were purchased from Taconic (Ry, Denmark). The mice were used at the age of 8–10 weeks. The mice had free access to water and to standard mouse chow (Altromin®) and were kept in a room with 12 h day/night cycle. All animal experiments were approved by The Danish Animal Inspectorate.

### Contact hypersensitivity

CHS experiments were performed largely as described previously [34]. In brief, the mice were sensitized on Day 0 by applying 20 µL 0.5% DNFB (1-fluoro-2.4-dinitrobenzene, Sigma, St Luis, MO) dissolved in 4:1 acetone (VWR, Radnor, PA)/olive oil (Sigma) on the shaved abdominal skin. Five days later, the baseline ear thickness on the left ear was measured after which both sides of the left ear were challenged by epicutaneous application of 20 μL 0.2% DNFB (10 μl on each side). The challenge treatment was performed under light anesthesia with isoflurane. The ear thickness of the left ear was measured at the indicated time-points after challenge with a dial thickness gauge from Mitutoyo (Mitutoyo Pocket Thickness Gages 7309; Kawasaki, Japan). Ear swelling (ΔT) was calculated as ear thickness 24, 48, or 72 h after challenge minus baseline ear thickness and was expressed as the mean ± standard error (SEM) in units of 10^−2^ mm. All groups always comprised at least five animals except in the cytokine/chemokine measurements seen in Figure 3C–F (4 mice/group) and in Figure 3G–I (8 mice/group).

### Antibody treatment

For depletion of neutrophils, mice were treated intraperitoneally (i.p) with rat anti-mouse Ly6G/C (Gr-1) monoclonal antibody (mAb), clone RB6.8C5 (BioXcell, West Lebanon, NH), rat anti-mouse Ly6G mAb, clone 1A8 (BioXcell) or their respective isotype controls rIgG2b (BioXcell) (for anti-Ly6G/C mAb clone RB6.8C5) and rIgG2a (BioXcell) (for anti-Ly6G mAb clone 1A8) in 1 mg/200 µL/mouse a day prior to challenge. Additionally, both anti-Ly6G mAb and anti-Ly6G/C mAb were tested in the doses 3 mg/mouse, 1 mg/mouse, or 0.25 mg/mouse. In the study where neutrophils were depleted at different time points, anti-Ly6G mAb-treatment was initiated at Day −6, Day −1, and Day 1 in three different groups, respectively. In the group that was depleted from Day −6 an additional dose was given at Day −1 and 1 to ensure sufficient depletion and in the group depleted from Day −1 an additional dose was given at Day 1. When examining the effect of blocking G-CSF, mice were treated with 0.5 mg/200 µL/mouse i.p. of rat anti-mouse G-CSF mAb (R&D Systems, Minneapolis, MN) or similar amounts of isotype control rIgG1 (BioXcell) 2 h prior to challenge as previously described [35]. A group of mice treated with 200 ul PBS was further included in both of the two mentioned studies. When testing the effect of blocking TNFα, rat-anti mouse TNFα mAb (BioXcell) or an appropriate isotype control rIgG1 (BioXcell) were injected at 0.5 mg/mouse i.p. in groups treated with 1 mg/mouse anti-Ly6G mAb, 1 mg/mouse rIgG2a or 200 µL PBS, respectively. All compounds were administered 1 day prior to challenge.

### Blood analysis to evaluate number of neutrophils in the peripheral blood

Blood samples (100 µL in EDTA coated eppendorf tubes (Eppendorph, Hamburg, Germany)) were collected from each mouse. The samples were first blocked with anti-CD32/CD16 (Fc block, BD Biosciences, San Jose, CA, USA) for 10 min and surface stained with the following anti-mouse mAb: CD8 APC (BDBiosciences), CD4 Qdot605 (Invitrogen, Carlsbad, CA), CD45 eFluor450 (eBiosciences, San Diego, CA), TCRβ PE-Cy7 (Biolegend, San Diego, CA), CD19 PerCPCy5.5 (BDBiosciences), CD88 PE (Biolegend), Ly6G/Ly6C FITC (clone RB6.8C5) (BDBiosciences), CD11b AF700 (eBiosciences). After staining, red blood cells were lysed with FACSlysing solution (BDBiosciences). Flow cytometric analysis of samples was analyzed on a BD LSRII flow cytometer equipped with a blue, red and violet laser and data was analyzed in BD FACS Diva software version 6.1.3. When both anti-Ly6G/C mAb (clone RB6.8C5) and anti-Ly6G mAb (clone 1A8) were included in the study (Figs. 4), neither anti-Ly6G nor anti-Ly6G/C were included as a neutrophil marker due to possible competition with the depleting antibodies. Thus, in Figures 4, neutrophils in the blood were expressed as the percentage of CD45^+^TCRB^−^CD19^−^CD11b^+^SSC^high^ cells of all CD45^+^ cells after gating for singlet-events by FSC and SSC and using a lymphocyte FSC/SSC size gate. It was further confirmed that the CD45^+^TCRB^−^CD19^−^CD11b^+^SSC^high^ cells were positive for CD88 (C5a receptor). To estimate an absolute number of neutrophils in the blood, BDTrucount beads (BD Biosciences) were included in the FACS analysis. When only the anti-Ly6G mAb (clone 1A8) or no depleting antibodies were included in the analysis neutrophils were gated as TCRB^−^CD19^−^CD11b^+^Ly6G/C+ and shown as either in % of CD45^+^ cells (Fig. 5) or given as absolute number (#) after using BDTrucount beads (BD Biosciences) (Figs. 7). When investigating if neutrophilia was present during the CHS response (Fig. 1) the neutrophil counts obtained from the flow cytometric analysis were confirmed by the use of a Medonic Hematology Analyzer (Boule Diagnostic, Stockholm, Sweden).

### Flow cytometry on ear infiltrating cells

To examine the cellular infiltrate in the ear 48 h after challenge, flow cytometric analysis was performed on infiltrating cells in the ear. Briefly, the inflamed ear was divided into dorsal- and ventral halves. Using a scalpel, the dermis was separated from epidermis and both parts were subsequently incubated with 2000 U/mL collagenase (Sigma) and 2000 U/mL DNAse (Roche, Basel, Switzerland) for 60 min. Next, ear tissue was passed through a 70 μm cell strainer before cells were washed and re-suspended in PBS (w/o Mg^2+^ and Ca^2+^, Gibco/Invitrogen). The cells were counted and cell suspensions were thereafter blocked with anti-CD32/CD16 (Fc block, BDBiosciences) for 10 min and stained with the following anti-mouse mAbs: CD8 APC (BDBiosciences), CD4 Qdot605 (Invitrogen), CD45 efluor450 (eBioscience), TCRβ PECy7 (Biolegend), CD19 PerCPCy5.5 (BDBiosciences), CD88 PE (Biolegend), Ly6G/Ly6C FITC (BDBiosciences), CD11b AF700 (eBiosciences) for 30 min. Flow cytometric analysis of samples was analyzed as described above.

### Flow cytometry on cells in the ear-draining lymph nodes

To examine the presence of CD8 T cells after challenge in the ear-draining lymph nodes the cervical superficial lymph nodes were removed 48 h post-challenge. Single-cell suspension was prepared by transferring the lymph node through a 70-μm cell strainer and washing cells with 1× phosphate-buffered saline (PBS) (w/o Mg2+ and Ca2 +; Gibco/Invitrogen). Cells were counted using a cell-counter and resuspended at 10^7^ cells/mL and 0.5 × 10^6^ cells/sample were used for staining. The cells were counted and blocked with anti-CD32/CD16 (Fc block, BDBiosciences) for 10 min and stained with the following anti-mouse mAbs: anti-mouse TCRb Qdot655 (Custom made), anti-mouse CD4 FITC (eBioScience) and anti-mouse CD8 Pacific Blue (BDBioscience) for 30 min. Flow cytometric analysis of samples was analyzed as described above.

### Cytokine measurements

Ears were removed 24 h after challenge and each ear was weighted and placed in 0.5 ml buffer (0.9% Saline with 0.01% Triton X-100 (Sigma) + 1 protease inhibitor cocktail tablet (Complete EDTA-free from Roche)) on ice. The biopsies were subsequently homogenized and centrifuged 15 min for 10,000*g* at 4°C. The supernatants were centrifuged once more before being frozen at −80° until use. Supernatants were analyzed with MILLIPLEX MAP Mouse Cytokine/Chemokine Panel (Millipore, Billerica, MA) by the Luminex detection method. Supernatants were analyzed for the following cytokines and chemokines: MIP-2 (CXCL2), MCP-1 (CCL2), KC (CXCL1), MIG (CXCL9), RANTES (CCL5), LIX (CXCL5), IL-4, IL-1β, IFNγ, IL-6, IL-10, G-CSF, and TNFα.

### Histology

Ears were excised from previously sensitized mice 24 h after challenge. They were subsequently fixed in 4% formalin, embedded in paraffin and sections were prepared and stained with hematoxylin and eosin.

### Detection of G-CSF in serum and tissue

Serum samples and homogenates prepared 0, 6, 10, 24, and 48 h after challenge in groups that were previous sensitized (+sensitized) or not (-sensitization) were analyzed for G-CSF using ELISA according to the manufacturer's recommendations (R&D Systems). Homogenates were prepared as described previously.

### Statistical analysis

Statistical analysis to assess differences between experimental groups was performed using a student *t*-test when comparing only two groups and a one-way ANOVA with Bonferroni's correction when comparing more than two groups. Differences were considered significant when *P* < 0.05.

## Abbreviations

CHS: contact hypersensitivity
DNFB: dinitrofluorobenzene
SSC: side scatter
PMN: polymorphonuclear
AUC: area under curve
